# Resolving the glycosaminoglycan signature of ischemic stroke brain using PRM-based IR-MALDESI mass spectrometry imaging

**DOI:** 10.1007/s00216-026-06334-3

**Published:** 2026-01-23

**Authors:** Tana V. Palomino, Noah Campbell, Yunxin Ouyang, Nidhi Naik, Adam M. Hawkridge, Tatiana Segura, David C. Muddiman

**Affiliations:** 1 Biological Imaging Laboratory for Disease and Exposure Research, Department of Chemistry, North Carolina State University, Raleigh, NC USA; 2 Department of Biomedical Engineering, Duke University, Durham, NC USA; 3 Department of Pharmaceutics, Virginia Commonwealth University, Richmond, VA USA

**Keywords:** Ischemic stroke, Chondroitin sulfate, Mass spectrometry imaging, IR-MALDESI, Parallel reaction monitoring

## Abstract

Stroke is the second most common cause of death in the world and a leading cause of disability. Ischemic stroke is the most common type of stroke (~87%), necessitating research into effective treatments. Chondroitin sulfate (CS) is a sulfated glycosaminoglycan (GAG) found in the central nervous system (CNS) that contains labile sulfate groups which, upon loss, leads to inaccurate structural annotations. Variable sulfation patterns have been implicated in several neurological diseases. Additionally, CS-GAG analysis is challenging due to labile sulfate groups and the presence of positional isomers. These isomers must be distinguished to develop effective targeted therapies. Currently, glycan mass spectrometry imaging (MSI) lacks soft ionization sources which impedes intact analysis of the labile sulfate modifications. Infrared matrix-assisted laser desorption electrospray ionization (IR-MALDESI) is a soft ambient ionization technique capable of preserving labile species without chemical derivatization. In this work, IR-MALDESI with parallel reaction monitoring (PRM) was used to energetically resolve and characterize intact mono-sulfated CS-GAG positional isomers in healthy and ischemic stroke brain. Our results revealed that both positional isomers were upregulated in the stroke brain and their relative abundance remained constant across the tissue.

**Graphical abstract:**

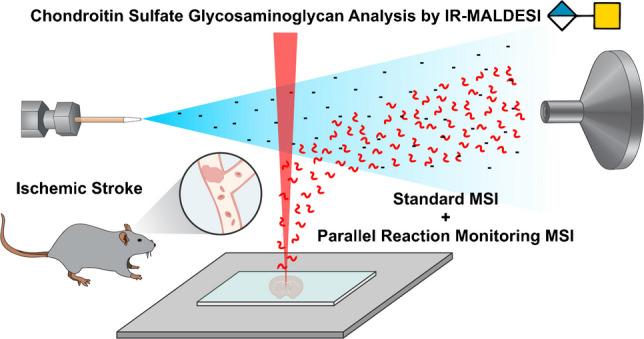

**Supplementary Information:**

The online version contains supplementary material available at 10.1007/s00216-026-06334-3.

## Introduction

**Fig. 1:**
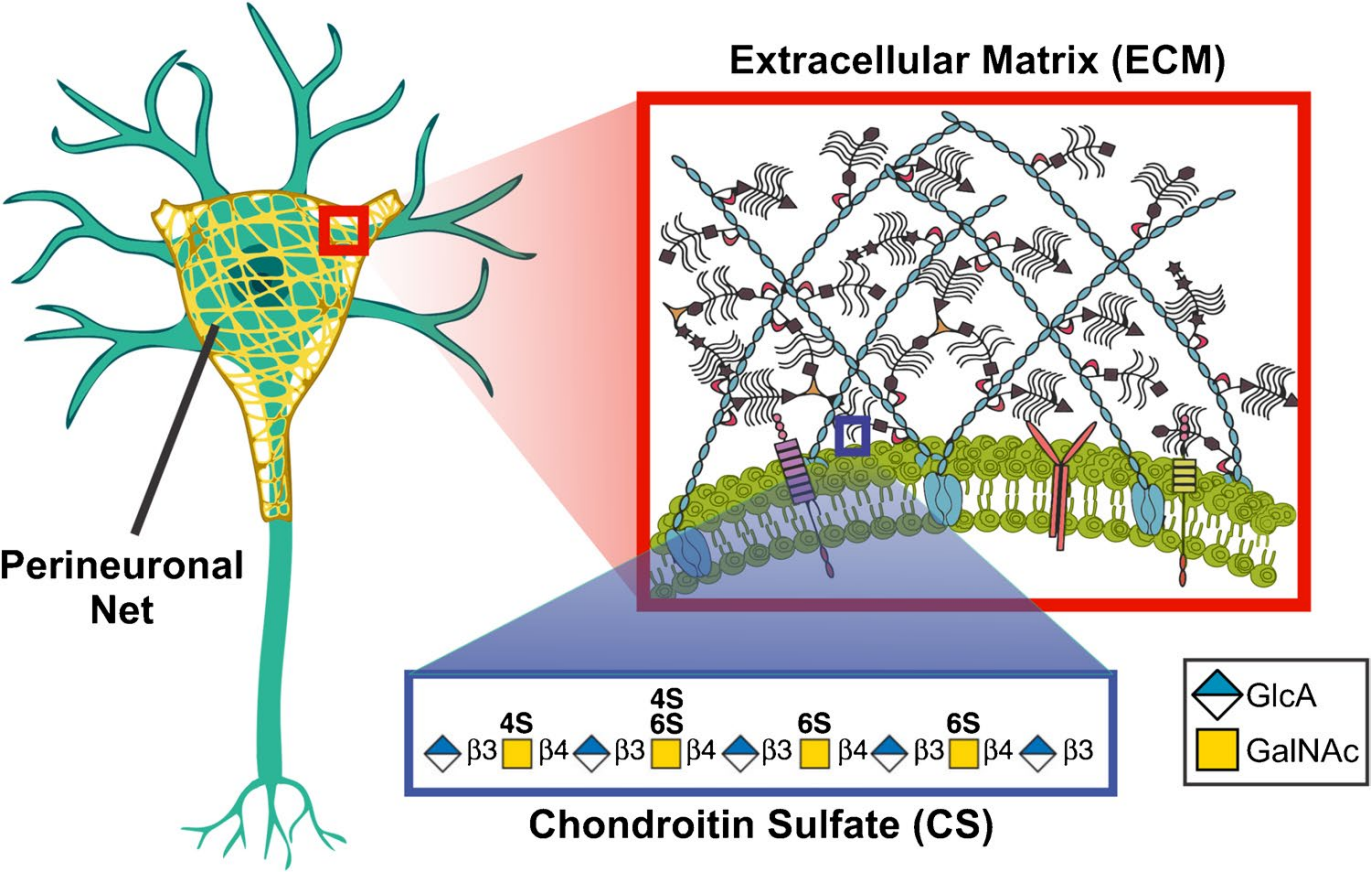
Chondroitin sulfate (CS) in the central nervous system (CNS). CS is a glycosaminoglycan (GAG) composed of repeating glucuronic acid (GlcA) and *N*-acetylgalactosamine (GalNAc) disaccharide units with varying degrees of sulfation. CS-GAGs make up a significant portion of the extracellular matrix (ECM) coating neurons in the form of perineuronal nets (PNNs)

Glycosylation is the most diverse post-translational modification (PTM) [1]. Glycosaminoglycans (GAGs) are a type of polysaccharide that post-translationally modify select proteins referred to as proteoglycans (PGs) that play important roles in the extracellular matrix (ECM) [2]. Chondroitin sulfate (CS) is a specific type of GAG that is composed of repeating glucuronic acid (GlcA) and *N*-acetylgalactosamine (GalNAc) disaccharide units with varying degrees of sulfation. The differential sulfation patterns facilitate protein interactions, particularly in the extracellular matrix (ECM), where they control biological processes related to chemotaxis, inflammation, receptor binding, and endocytosis. CS-GAGs are heavily involved with signaling in the central nervous system (CNS) ECM [3]. Within the CNS ECM, interneurons are wrapped with perineuronal nets (PNNs) which play significant roles in synaptic plasticity, memory, and disease [4–9]. One of the main components of PNNs is CS proteoglycans (CSPGs) such as aggrecan, neurocan, and brevican [10]. A schematic diagram depicting the CS-bound PGs in PNNs is shown in Fig. 1.

**Fig. 2:**
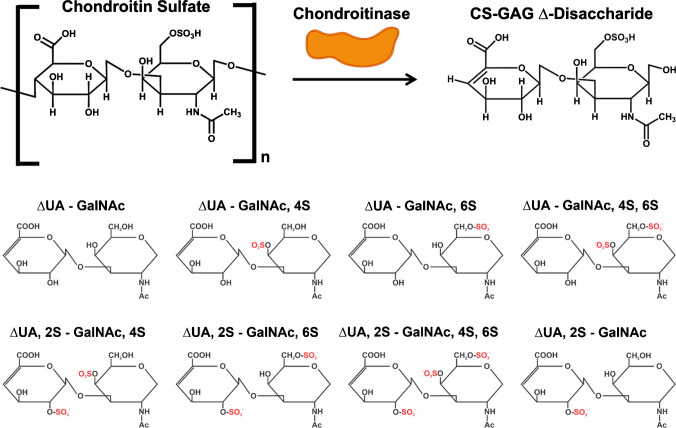
Enzymatic digestion of chondroitin sulfate with chondroitinase yields ∆-disaccharides. The disaccharides can vary between non-, mono-. di-, and tri-sulfated structures. UA, uronic acid; GalNAc, *N*-acetylgalactosamine; S, sulfate

Altered sulfation in CS-GAGs has been implicated in several neurological diseases using techniques such as LC-MS/MS, yet the spatial location of these changes remains unresolved [5, 7, 9, 11–13]. A common first step in analyzing polydisperse CS oligosaccharides with molecular weights up to 100 kDa is to enzymatically break down the large polymer into disaccharide units using chondroitinase ABC. By digesting the CS-GAGs into smaller units, their disaccharide composition can be determined quantitatively (Fig. 2) [14]. The eight possible CS-GAG disaccharide structures (Fig. 2) contain differential sulfation at the C-4 and C-6 positions of GalNAc, and the C-2 position of GlcA [15]. These structures are β1–3 linked, but they can also exist as amine-acid disaccharides that are β1–4 linked, totaling 16 theoretical CS-GAG disaccharides [15]. The biologically relevant structures that will be of focus in this work are mono-sulfated ∆UA-GalNAc, 4S (∆4S-CS) and ∆UA-GalNAc, 6S (∆6S-CS) [16].

Stroke is a leading cause of disability and the second most common cause of death in the world [17, 18]. Upregulation of CS-GAGs has been found in stroke samples. However, the specific details about which positional isomers are upregulated and where within a tissue section remain unknown. Most importantly, differentiating between biologically relevant mono-sulfated positional isomers is essential for determining specific stroke-induced changes. Therefore, we applied parallel reaction monitoring (PRM) MSI to distinguish mono-sulfated positional isomers across both healthy and stroke mouse brain [19–21]. PRM is a method in which high-resolution accurate-mass (HRAM) spectrometry is used to accurately quantify specific ions in a sample. A precursor ion is isolated in the quadrupole and passes through the C-trap before entering the Higher-Energy Collisional Dissociation (HCD) cell. Normalized collision energy (%NCE) is then applied to the HCD cell to produce fragment ions. All fragment ions then re-enter the C-trap before being ejected into the Orbitrap mass analyzer for detection. This method allows for all fragment ions to be detected in one experiment, and therefore the spatial distribution of all fragment ions can be visualized.

Infrared matrix-assisted laser desorption electrospray ionization (IR-MALDESI) is a soft ionization technique that combines the benefits of MALDI and electrospray ionization (ESI) [22]. The softness of this source allows for the detection of intact labile carbohydrates without the use of chemical derivatization. As a result, this method has been used to analyze sialylated *N*-linked glycans and sulfated GAGs [23–25]. Since the sulfate groups on GAGs are labile, it is important to use a technique that does not deposit large amounts of internal energy that could cause loss. Previous work showed that ∆4S-CS and ∆6S-CS positional isomers can be differentiated using MS/MS since distinct fragments are produced from each isomer [16]. Therefore, in this work, we expand upon this principle and use PRM MSI by IR-MALDESI to energetically resolve mono-sulfated positional isomers in healthy and stroke brain. IR-MALDESI’s ability to spatially resolve these isomers provides the spatial context necessary for accurate quantification of stroke-induced changes.

## Methods

### Ischemic stroke model

**Fig. 3:**
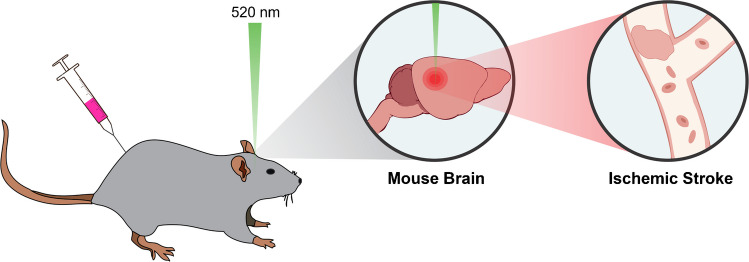
Ischemic stroke model in mouse brain. A photoactive dye is injected intraperitoneally into the mouse. Upon laser fire, dye activation causes coagulation and induces the ischemic stroke

Male mice 8–12 weeks of age provided by the Segura Lab at Duke University Department of Biomedical Engineering were prepared according to the Institutional Animal Care and Use Committee (IACUC). A schematic depicting the ischemic stroke model is shown in Fig. 3 [26]. The mice are anesthetized before drilling a small hole on top of their skull. Rose Bengal dye is a photoactive dye that is then injected intraperitoneally. A laser firing at 520 nm is focused at a stereotaxic coordinate 1.0 mm anterior and 1.5 mm lateral (left) relative to bregma, targeting the M1 motor cortex. After irradiation, the excitation causes oxygen radicals to form and induce endothelial damage and coagulation, causing the photothrombotic stroke. After 5 days, healthy and stroke mouse brains were harvested and processed into formalin-fixed paraffin-embedded (FFPE) tissue blocks using established protocols [27].

### Formalin-fixed paraffin-embedded tissue sample preparation

All chemicals were obtained from Sigma-Aldrich unless noted otherwise. FFPE tissue blocks were sectioned into 5-µm-thick sections and mounted onto charged slides. Three technical replicates each for healthy and stroke were prepared using established protocols [28]. The slides were heated at the bottom of a 60-°C oven for 1 h to melt paraffin wax. The tissues were then subjected to a series of washes consisting of xylene (2 × 3 min), 95% ethanol (1 × 1 min), 70% ethanol (1 × 1 min), and water (2 × 3 min) to dewax/delipidate and rehydrate the tissue sections. Slides were dried in a vacuum desiccator for 5 min before performing antigen retrieval. The samples were submerged in a slide mailer containing citraconic acid buffer (controlled to pH 3 using 12 M HCl) prepared fresh the same day and placed in a vegetable steamer (Rival) for 30 min to undo protein crosslinks formed during formalin fixation. The slides were then slowly rehydrated with water before enzyme spraying. Chondroitinase ABC was prepared as a 1 mg/mL concentration in 50 mM ammonium acetate solution controlled to pH 8 with sodium hydroxide and pneumatically sprayed onto tissue sections using a TM-Sprayer (HTX Technologies, Carrboro, NC). The samples were incubated in humidity chambers with relative humidity > 95% for 5 h at 37 °C. A healthy and stroke section not used for the experiment were stained using Histogene blue staining solution and Permount mounting solution to reveal tissue morphology.

### IR-MALDESI analysis

Mass calibration was performed each day of analyses. A solution consisting of 60% ACN and 1 mM acetic acid was used as the electrospray solvent. Stable electrospray was achieved using 3100 V and a flow rate of 1.5 µL/min. An IR laser firing at a wavelength of 2970 nm (JGM Associates Burlington, MA, USA) and 10 pulses per burst was used. A laser energy of 3 mJ was achieved. The spot size and step size used for all samples was 150 µm. Tissue sections were placed onto a Peltier-cooled stage inside of a humidity-controlled chamber to facilitate the formation of an ice matrix over the sample. The IR laser resonantly excites the water molecules present endogenously in the sample and exogenously in the ice matrix. Approximately 115 mm^3^ of tissue was ablated at each pixel/laser fire. The desorbed neutrals partition into the ESI droplets and are post-ionized before entering the mass spectrometer for detection. IR-MALDESI is coupled to an Orbitrap Exploris 240 mass spectrometer (Thermo Fisher Scientific, Bremen, Germany) where automatic gain control (AGC) was turned off and the injection time was set to 90 ms.

### Parallel reaction monitoring

**Fig. 4:**
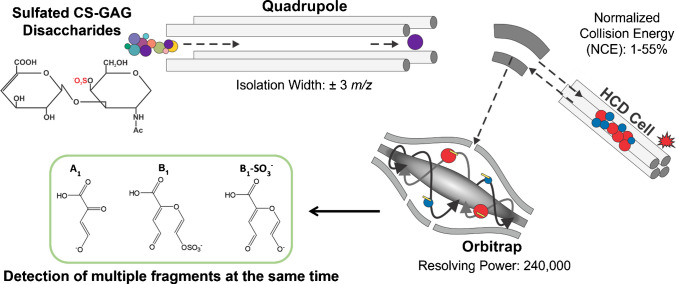
Parallel reaction monitoring mass spectrometry imaging (PRM MSI). Sulfated disaccharides are isolated in the quadrupole before entering the C-trap. The ions of interest enter the HCD cell and are fragmented at a user-defined normalized collision energy (NCE) before returning to the C-trap and ejection into the Orbitrap for detection. This technique allows for the detection of all fragments in one simultaneous run, and the spatial distribution of the fragment ions across a tissue section can be visualized

The PRM MSI experiment is shown in Fig. 4. PRM MSI allows for the detection of all fragment ions from one run. The ions of interest were isolated in the quadrupole within a ± 3-*m*/*z* window and pass through the C-trap before being fragmented in the HCD cell. Fragment ions then re-enter the C-trap before being ejected into the Orbitrap for detection. For the energy breakdown curves, mono-sulfated ∆4S-CS and ∆6S-CS disaccharide standards were obtained (Galen Molecular, North Haven, CT, USA) to determine the % normalized collision energy (NCE) optimal for PRM MSI. Each standard was prepared to a concentration of 100 µM. The precursor *m*/*z* of interest for both standards was 458.0610 *m*/*z*, which corresponds to the singly deprotonated ion, or [M-H^+^]^−^. 25 scans were recorded and averaged for each % NCE between 1 and 55%. 30% NCE was selected for PRM MSI experiments to maximize fragment ion signal.

## Results and discussion

The purpose of this study was to implement the ischemic stroke mouse model to create a lesion in the brain that mimics stroke to further our understanding of the GAG signature in the brain in a spatial manner. FFPE healthy and stroke brain was dewaxed/delipidated and rehydrated before antigen retrieval. Enzymatic digestion with chondroitinase yields CS-GAG ∆-disaccharides. The samples were analyzed in negative mode MS1 and MS2 IR-MALDESI to determine the spatial distribution of non- and mono-sulfated CS-GAG disaccharides within healthy and stroke mouse brain. Stained tissue sections revealed the location of the stroke lesion in ischemic stroke brain. Sequential paired covariance (SPC) was used to improve visualization of the stroke lesion. SPC also revealed the upregulation of CS-GAGs in the glial scar region of the mouse brain. Parallel reaction monitoring (PRM) MSI was implemented to differentiate between mono-sulfated positional isomers (4S-CS and 6S-CS). Standards were used to optimize %NCE for use in imaging experiments. PRM MSI determined that both positional isomers were upregulated in the stroke region. Both standard MSI and PRM MSI provide distinct benefits for the analysis of CS-GAG disaccharides.

**Fig. 5:**
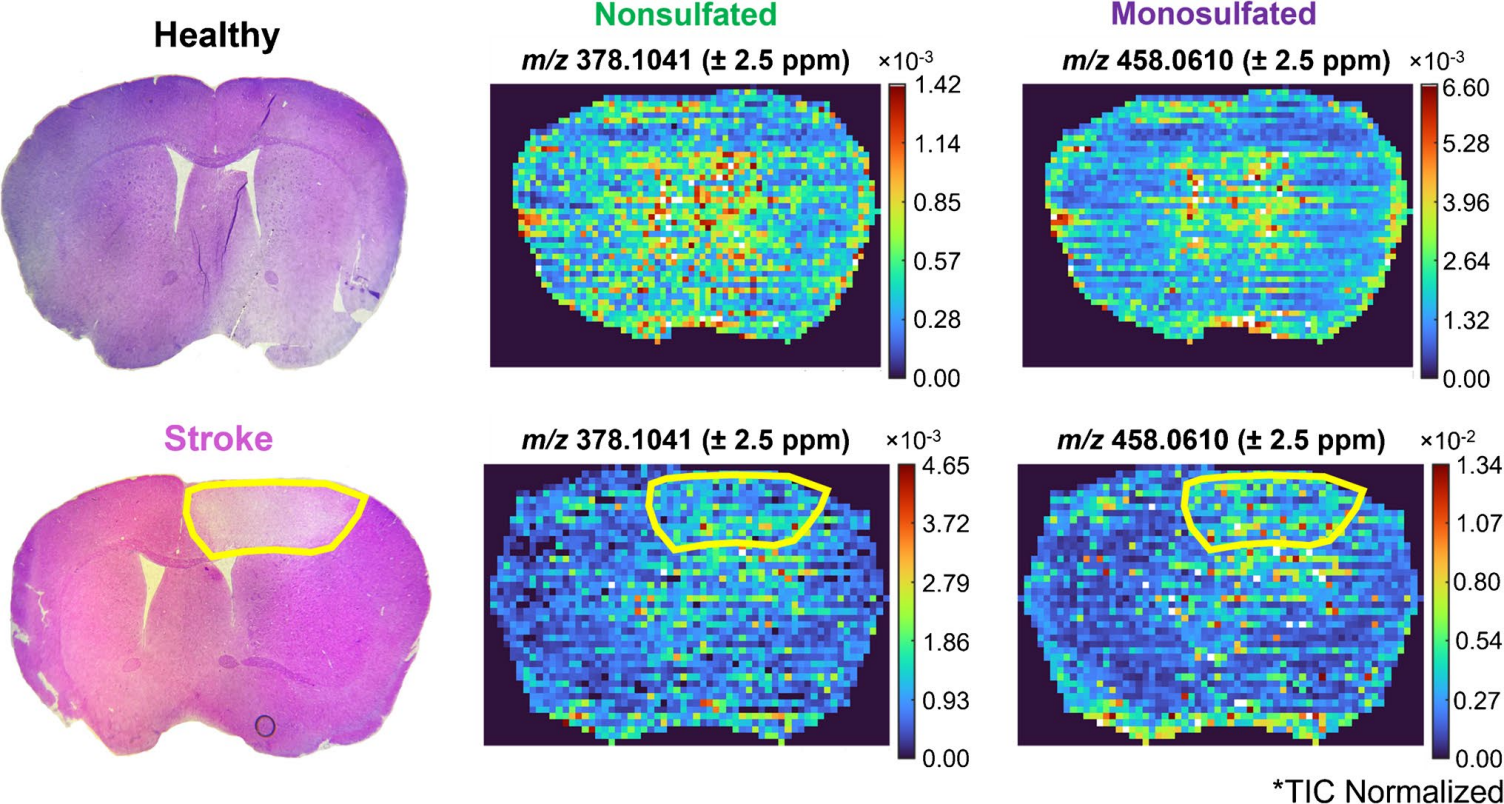
Non- and mono-sulfated CS-GAG disaccharides in healthy brain (top) and stroke brain (bottom). Data was normalized to the total ion current (TIC). The ischemic stroke lesion is outlined in yellow. Upregulation of both non- and mono-sulfated CS-GAGs was detected in stroke. Non-sulfated disaccharides increased by a factor of 3, and mono-sulfated disaccharides increased by a factor of 2

Non- and mono-sulfated CS-GAG disaccharides were detected in healthy and stroke brain as singly deprotonated ions in all replicate samples within a 2.5-ppm mass measurement accuracy (MMA) (Figs. 5, S1). 378.1041 *m*/*z* corresponded to the non-sulfated [M-H^+^]^−^ peak, and 458.0610 *m*/*z* corresponded to the mono-sulfated [M-H^+^]^−^ peak. All data was normalized to the total ion current (TIC) to improve data visualization. Sodium adducts for the mono-sulfated disaccharide were observed at low abundance and therefore not included in this analysis. Consistent with previous work, loss of the sulfate group within mono-sulfated disaccharides was not observed [25]. Non-sulfated disaccharides in the entire brain increased by a factor of 3 in stroke, and mono-sulfated disaccharides increased by a factor of 2. Mass spectra comparing healthy and stroke brain regions are shown in Figure S2.

**Fig. 6:**
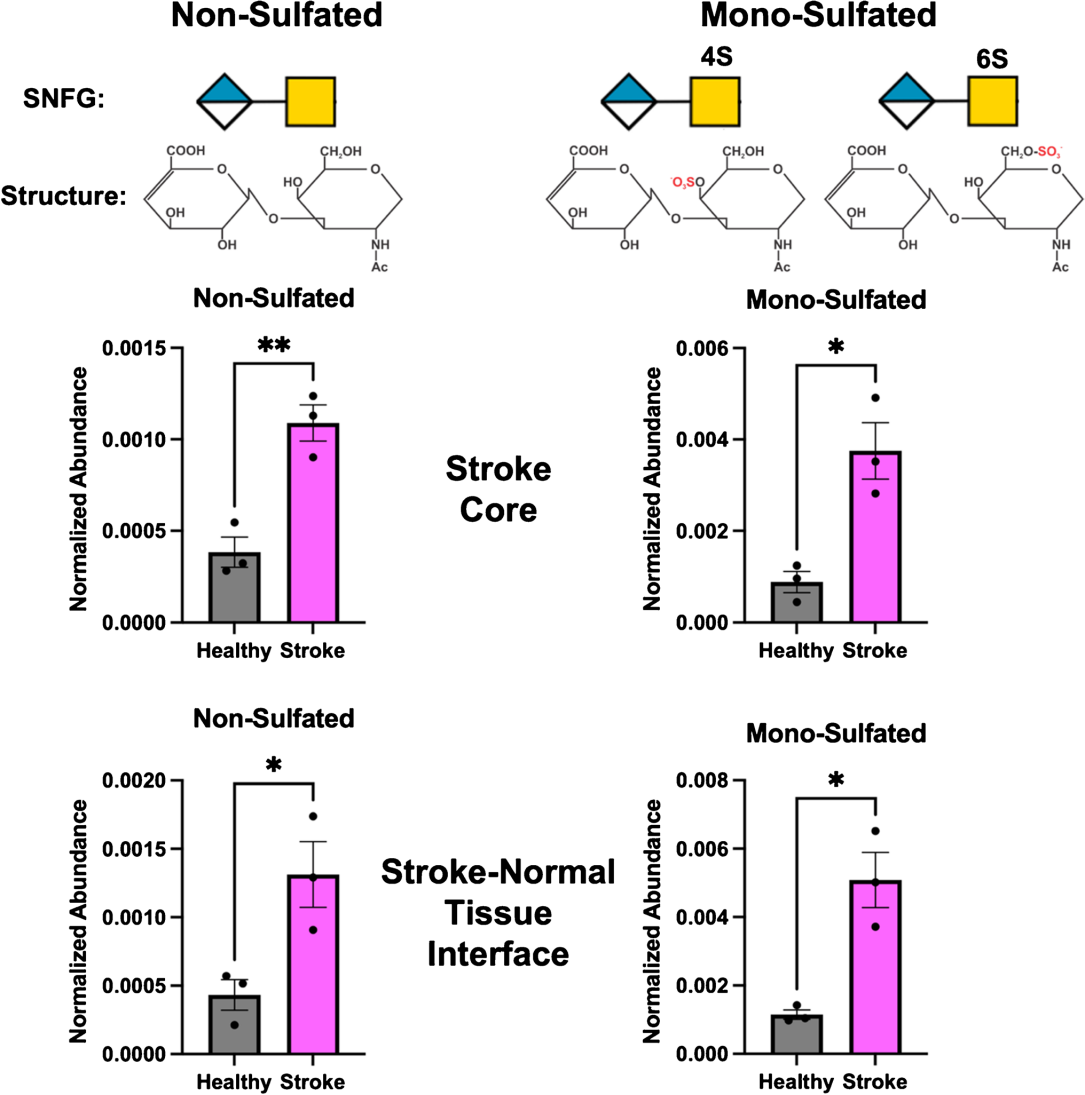
Non- and mono-sulfated disaccharides were upregulated in the stroke region of the brain. Statistical significance was calculated using an unpaired *T*-test (*p < 0.05, **p < 0.01). Symbol nomenclature for glycans (SNFG) and structure for each disaccharide are displayed. Mono-sulfated disaccharides can exist as two isomers (∆4S-CS and ∆6S-CS)

Stained tissues revealed the stroke region in the brain, allowing for the outline of the stroke lesion to be drawn over the ion heatmaps and for use in spatial analysis. A region of interest (ROI) was drawn within the stroke lesion core and the stroke-normal tissue interface. This ROI was then placed over the same regions in the healthy brain ion heatmap. Abundance values for non- and mono-sulfated disaccharides were extracted from these ROIs to determine statistical significance using an unpaired *t*-test with 0.05 significance and all three technical replicates. Both detected disaccharides were significantly upregulated in the stroke core region of the brain and the stroke-normal tissue interface (Fig. 6). Disaccharide structures are displayed along with their corresponding Symbol Nomenclature for Glycans (SNFG). The possible biologically relevant mono-sulfated positional isomers, ∆4S-CS and ∆6S-CS, are listed. Since differentiating these positional isomers may help inform stroke treatment regimens, future studies will focus on developing these methods for MSI.

**Fig. 7:**
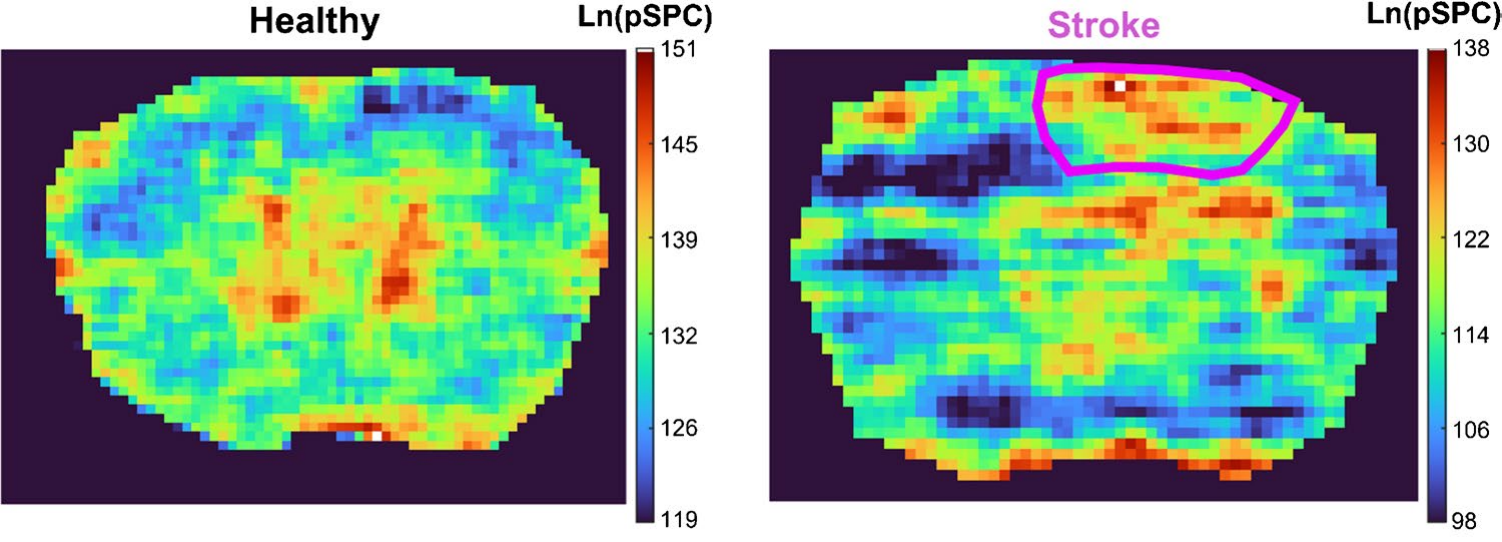
Sequential paired covariance (SPC) elucidates mono-sulfated CS-GAG localization in stroke brain. The stroke lesion is outlined in purple. SPC is the dot product of neighboring pixels and reduces the influence of variable noise peaks. This significantly improved the visualization of the stroke lesion without affecting the relative abundance of the data

To further improve the visibility of tissue features, sequential paired covariance (SPC) was performed on the mono-sulfated CS-GAG ion heatmap (Fig. 7) [29]. SPC represents the dot product of neighboring pixels and improves the appearance of ion heatmaps by reducing the effect of varying noise peaks. For this spatial analysis, the natural log of the dot product SPC was plotted for both healthy and stroke tissues. The main benefit of the SPC algorithm is that it does not change the relative abundance of the raw data, allowing for biologically accurate comparison between healthy and stroke brain. SPC not only improved the visibility of the upregulation in the stroke lesion, but also in other regions of the tissue, allowing for further investigation into the changes in tissue morphology. One of the areas in which stroke-induced changes to CS-GAGs were observed was in the ventricles of the brain. Interestingly, a decrease in CS-GAGs was observed in the ventricles in stroke brain compared to healthy. This could be attributed to different factors such as degradation and altered CSF fluid dynamics. However, further research is needed into this observation to fully understand the changes in CS-GAGs’ signature in the ventricle region after ischemic stroke.

**Fig. 8:**
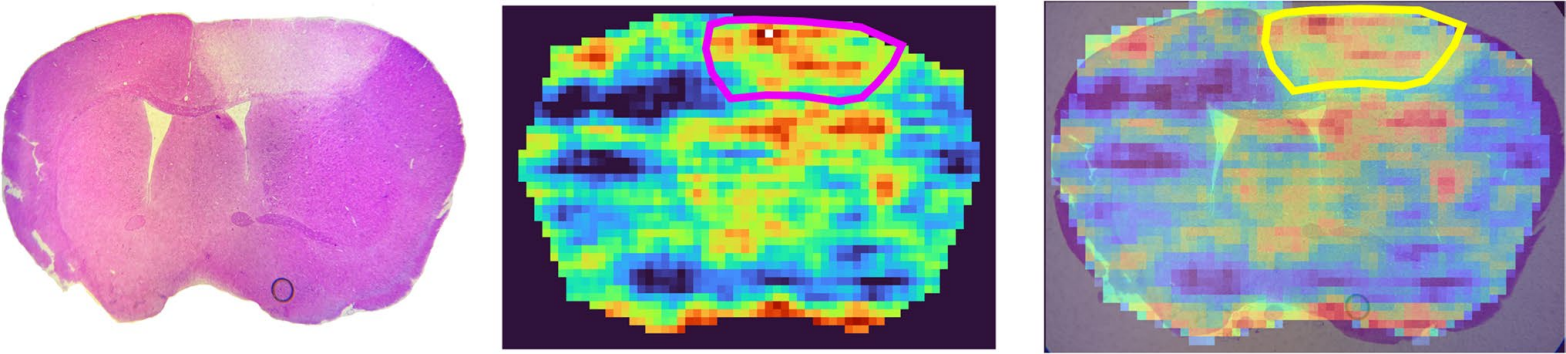
Image overlay (right) of stained stroke brain (left) and mono-sulfated CS-GAG disaccharide SPC heatmap (middle). Upregulation of mono-sulfated CS-GAG localizes to the stroke lesion in ischemic stroke brain

Upregulation was also observed outside of the stroke lesion, which corresponds to the formation of a glial scar. The glial scar is mainly composed of CSPGs and is formed as a response to damage in the CNS, acting as a barrier to prevent the wound from spreading further into the brain [30, 31]. An image overlay of the mono-sulfated SPC and stained ischemic stroke brain is shown in Fig. 8, which further highlights the ischemic stroke lesion and the glial scar formed right outside of the lesion. The results detailed in this study are consistent with previous studies in which upregulation of CSPGs is observed after stroke [32]. However, this upregulation serves a dual role. While it is initially essential for preventing further damage to the CNS, the presence of CSPGs can also inhibit axonal regeneration and recovery [33, 34, 35]. These results ultimately help further our understanding of stroke-induced damage in order to help us develop adequate treatment regimens.

**Fig. 9:**
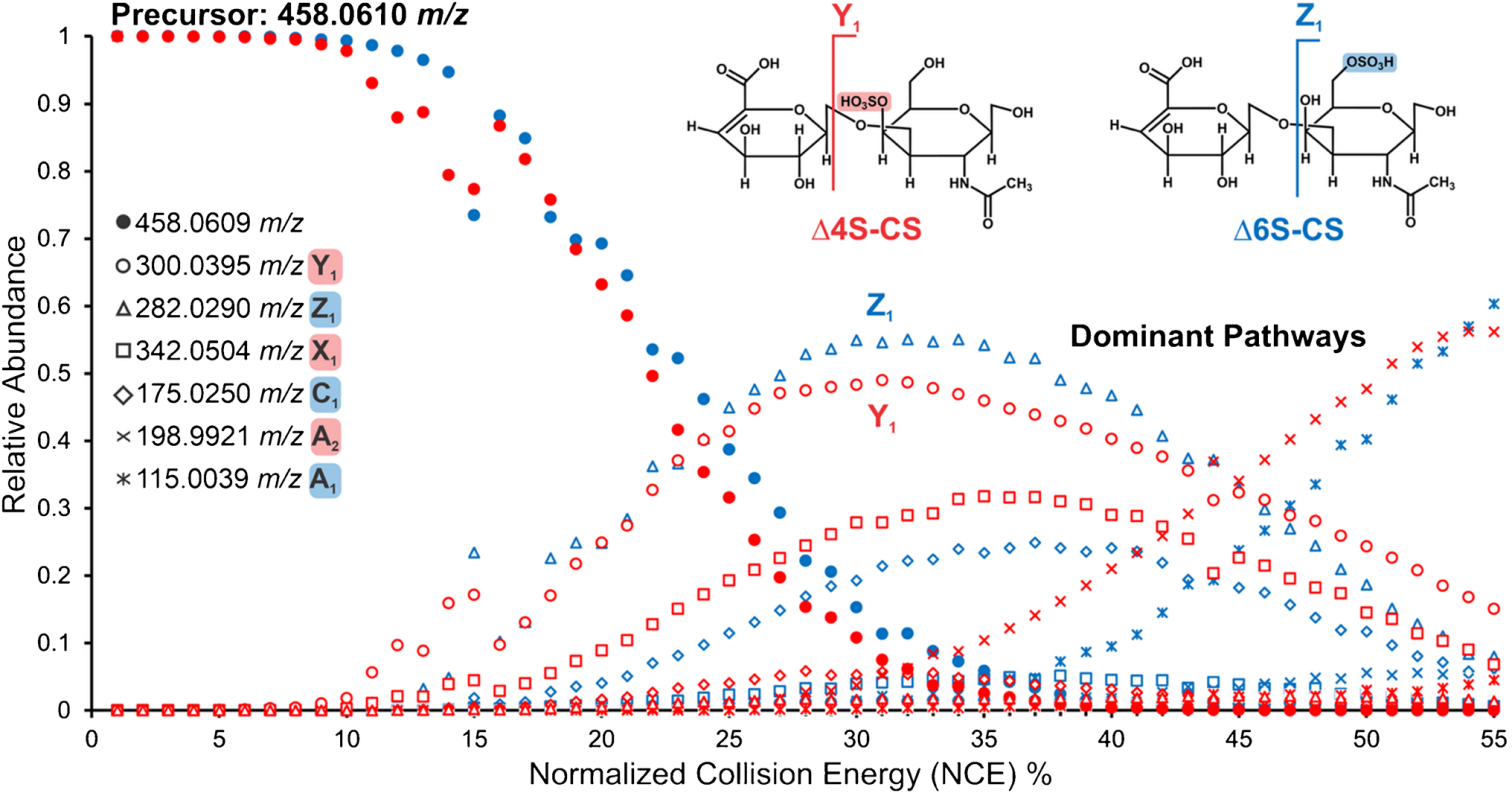
Energy breakdown curves for ∆4S-CS (red) and ∆6S-CS (blue) mono-sulfated positional isomers. Each shape corresponds to different fragment ions, and the color represents which positional isomer had the dominant abundance. Distinct fragmentation patterns were observed for both positional isomers. The dominant pathways for ∆4S-CS and ∆6S-CS are Y_1_ and Z_1_, respectively

To investigate differences in upregulation between sulfated positional isomers, this study implemented PRM MSI. Energy breakdown curves for mono-sulfated ∆4S-CS and ∆6S-CS were developed to determine the optimal % NCE for PRM MSI of healthy and stroke tissues (Fig. 9). Both precursor ions had an *m*/*z* ratio of 458.0610. The same six fragment ions were observed for each standard; however, the relative abundances of each fragment vastly differed between the positional isomers. The dominant pathway for ∆4S-CS produces the Y_1_ fragment, while the dominant pathway for ∆6S-CS produces the Z_1_ fragment. Additionally, ∆4S-CS also had significantly more X_1_ and A_2_ fragments, while ∆6S-CS had more C_1_ and A_1_ fragments. The energy at E_1/2_ was approximately 23% NCE, and at this energy, completely distinct fragments were observed between ∆4S-CS and ∆6S-CS, despite the only structural difference being the position of the sulfate group within the disaccharide. This indicates that MS/MS can be used to energetically resolve mono-sulfated positional isomers and can be applied to MSI studies investigating disease. 30% NCE was selected for PRM MSI of mono-sulfated CS-GAG disaccharides to maximize fragment ion signal.

**Fig. 10:**
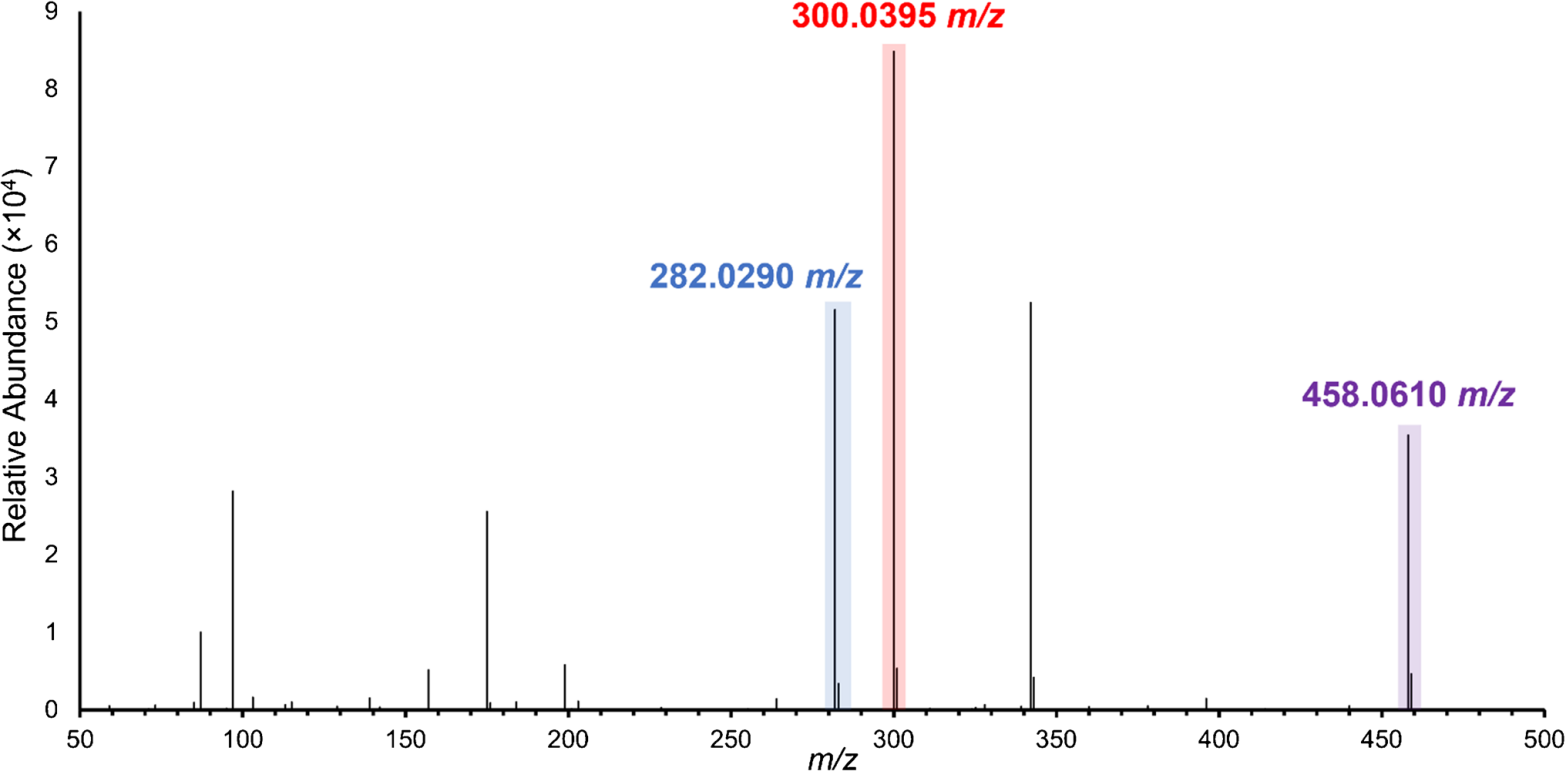
Equimolar mixture of ∆6S-CS and ∆4S-CS fragmented at 30% NCE reveals a 3:5 ratio of the ∆6S-CS dominant peak to the ∆4S-CS dominant peak. 282.0290 *m*/*z* is the ∆6S-CS dominant pathway, 300.0395 *m*/*z* is the ∆4S-CS dominant pathway, and 458.0610 *m*/*z* is the precursor ion

Before performing imaging experiments, we needed to determine if there were any differences in fragmentation and ionization efficiencies between ∆4S-CS and ∆6S-CS. This difference would affect the relative abundance of observed fragments when performing MS/MS on 1 *m*/*z* where the relative abundance of each isomer is unknown. Therefore, an equimolar mixture of ∆4S-CS and ∆6S-CS was fragmented using 30% NCE (Fig. 10). This revealed a 3:5 ratio of the ∆6S-CS dominant fragment to the ∆4S-CS dominant fragment. Since this is not a 1:1 ratio, this indicates that a correction factor would need to be implemented to the PRM MSI data during analysis to account for differences in fragmentation and ionization efficiencies between ∆4S-CS and ∆6S-CS and accurately quantify the relative abundance of these structural isomers.

**Fig. 11:**
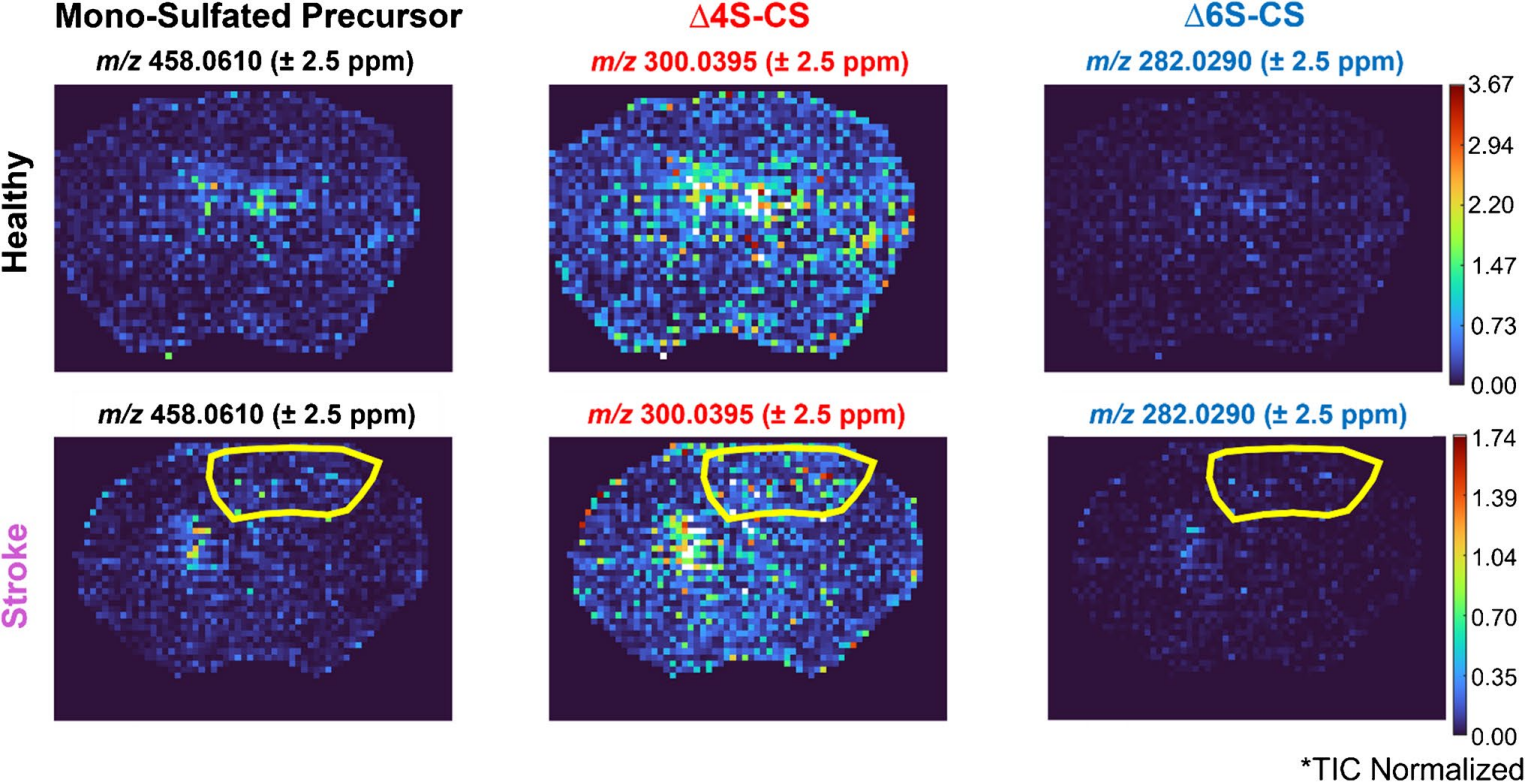
Parallel reaction monitoring (PRM) mass spectrometry imaging (MSI) of mono-sulfated CS-GAGs in healthy (top) and stroke (bottom) brain. The stroke lesion is outlined in yellow. Data was normalized to the total ion current (TIC); 30% normalized collision energy (NCE) was used to maximize fragment ion signal. In both healthy and stroke brain, the ∆4S-CS (red) positional isomer was an order of magnitude more abundant than the ∆6S-CS (blue) positional isomer

**Fig. 12:**
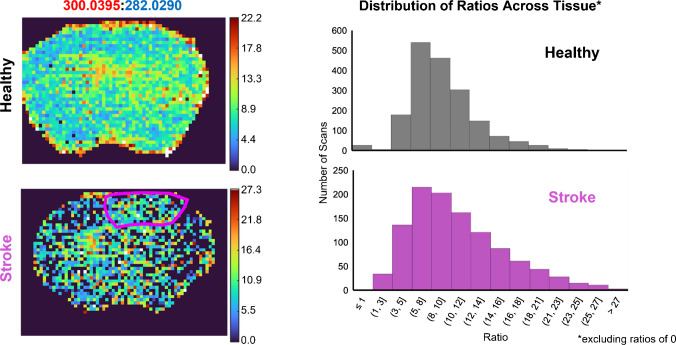
The ratio of the ∆4S-CS dominant fragment (300 *m*/*z*) to the ∆6S-CS dominant fragment (282 *m*/*z*) was plotted in healthy and stroke brain to visualize upregulation of any specific positional isomers. Histograms of the ratios across the tissue were plotted to determine consistency in upregulation between healthy and stroke brain positional isomers and excluded ratios of zero. Similar distributions between healthy and stroke histograms indicate consistent upregulation of both mono-sulfated positional isomers detected across stroke brain

The results for PRM MSI of mono-sulfated CS-GAGs in healthy and stroke mouse brain are shown in Fig. 11. Both ∆4S-CS and ∆6S-CS positional isomers were detected in healthy and stroke within a 2.5-ppm mass measurement accuracy (MMA). The ∆4S-CS positional isomer was an order of magnitude higher in abundance than the ∆6S-CS positional isomer in both healthy and stroke, which is consistent with previous work [16]. To determine whether a specific positional isomer was more upregulated in stroke, the ratio of the ∆4S-CS dominant peak to the ∆6S-CS dominant peak was plotted across the entire mouse brain (Fig. 12). A histogram of these ratios for healthy and stroke was also plotted to better visualize any differences. Interestingly, a consistent distribution of the ratios was observed across healthy and stroke. Despite lower sensitivity detected in the stroke brain, these similar distributions indicate a consistent upregulation of both mono-sulfated positional isomers in stroke.

While standard MSI provides important information for a discovery-based study aiming to broadly identify ions in a sample, PRM MSI is a targeted analysis that aims to resolve the isomers for one precursor ion of interest. In the case of CS-GAGs, mono-sulfated disaccharides can exist as multiple isomers and require PRM MSI to distinguish them. A further comparison between standard and PRM MSI is detailed in Table 1. Both methodologies provide distinct benefits and are extremely useful depending on the context of the study.

In this work, the CS-GAG signature of ischemic stroke brain was elucidated using IR-MALDESI MSI and PRM imaging. The upregulation of both mono-sulfated positional isomers was detected in ischemic stroke brain, particularly in the stroke lesion and glial scar. This is the first study to use PRM MSI and energetically resolve sulfated GAG isomers using MSI. The development of this methodology would not have been possible without the inherent softness of IR-MALDESI that detects accurate spatial information without loss of labile sulfate groups. Biologically relevant standards were directly analyzed and confirmed that only negligible loss of sulfate occurred (Figures S3, S4), and this was further evaluated in a previous study [25]. Our work herein lays the groundwork for investigating different time points post-stroke to monitor the change in GAG signature as the stroke wound heals and determine if different isomers are upregulated or downregulated over time. GAG-based therapeutic strategies are currently being developed, and this methodology can ultimately help further the development of these promising therapies by targeting specific isomers associated with ischemic stroke.

**Table 1:** Comparison of standard and PRM MSI

	Standard MSI	PRM MSI
Purpose	Discovery based	Targeted analysis
Raw data	Full mass spectrum with all detected ions	Fragmentation spectrum with 1 precursor ion window and all corresponding fragment ions
Isomer differentiation	Cannot differentiate between isomers	Can energetically resolve isomers using MS/MS
Heatmaps	Visualization of the spatial distribution of all detected ions	Visualization of the spatial distribution of all isomers corresponding to one precursor ion
Statistical analysis	Relative quantification of all detected ions	Can be performed after accounting for differences in fragmentation and ionization efficiency between isomers
Instrumentation	Requires a mass spectrometer with MS1 capabilities	Requires a mass spectrometer with MS/MS capabilities

## Conclusions

IR-MALDESI is a soft atmospheric pressure ionization as no fragmentation of sulfated CS-GAGs was observed. The ischemic stroke model detected significant upregulation of non- and mono-sulfated CS-GAGs in the stroke lesion in the brain and in the glial scar. PRM MSI was implemented using IR-MALDESI to energetically resolve mono-sulfated CS-GAG positional isomers. Using CS-GAG disaccharide standards, the fragmentation pathways for ∆4S-CS and ∆6S-CS were defined; Y_1_ and Z_1_ were the dominant pathways, respectively. PRM MSI revealed consistent upregulation of both mono-sulfated CS-GAG positional isomers in the stroke region, and their relative abundance remained constant. This work can ultimately help facilitate the development of novel therapies to help patients that suffered from ischemic stroke.

## Supplementary Information

Below is the link to the electronic supplementary material.

Supplementary file1 (DOCX 1.28 MB)

## Data Availability

The dataset used in this study is available at 10.5061/dryad.qnk98sfxf.
