# Supplementary material for: Resolving the glycosaminoglycan signature of ischemic stroke brain using PRM-based IR-MALDESI mass spectrometry imaging
Source: Anal Bioanal Chem. 2026 Jan 23;418(7):2083–94. doi: 10.1007/s00216-026-06334-3 (PMC12999702; doi:10.1007/s00216-026-06334-3)
Supplement: Supplementary file 1 — Supplementary file1 (DOCX 1.28 MB) [file 216_2026_6334_MOESM1_ESM.docx]

**Supporting Information**

**Resolving the Glycosaminoglycan Signature of**

**Ischemic Stroke Brain Using PRM-Based IR-MALDESI Mass Spectrometry Imaging**

Tana V. Palomino^1^, Noah Campbell^2^, Yunxin Ouyang^2^, Nidhi Naik^3^, Adam M. Hawkridge^3^,Tatiana Segura^2^ and David C. Muddiman^1^*

*^1^Biological Imaging Laboratory for Disease and Exposure Research, North Carolina State University, Raleigh, NC, USA*

*^2^Department of Biomedical Engineering, Duke University, Durham, NC, USA*

*^3^Department of Pharmaceutics, Virginia Commonwealth University, Richmond, VA, USA*

**Submitted to:** *Analytical and Bioanalytical Chemistry*

**Submitted**: August 28^th^, 2025

**Revised:** November 20, 2025

**Manuscript**: 24 Pages / 13 Figures / 1 Table / 4 Supplemental Figures

**Keywords**: Ischemic Stroke, Chondroitin Sulfate, Mass Spectrometry Imaging, IR-MALDESI, Parallel Reaction Monitoring

***Author for Correspondence**

David C. Muddiman, Ph.D.

Biological Imaging Laboratory for Disease and Exposure Research

Department of Chemistry

North Carolina State University

Phone: 919-513-0084

Email: [dcmuddim@ncsu.edu](mailto:dcmuddim@ncsu.edu)


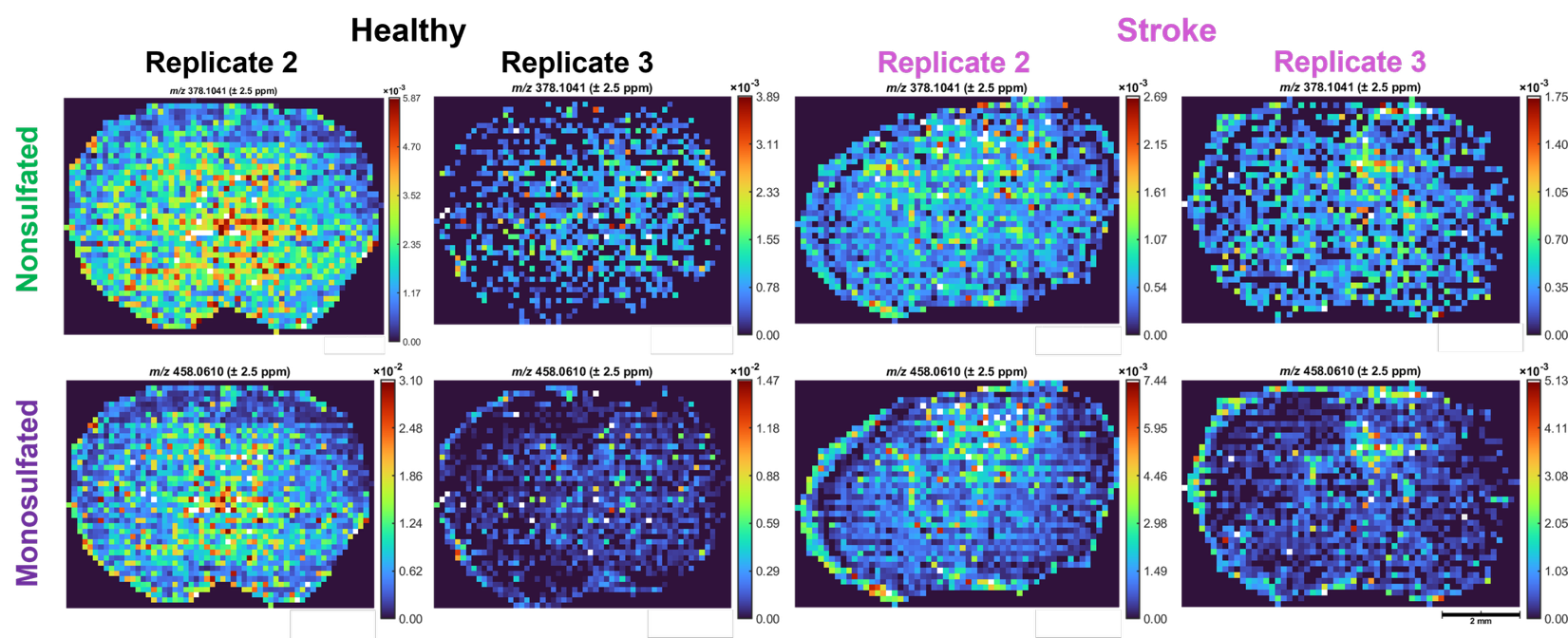


**Figure S1**. Technical replicates 2 and 3 for healthy and stroke brains. Non- and mono-sulfated disaccharides were detected in each replicate.


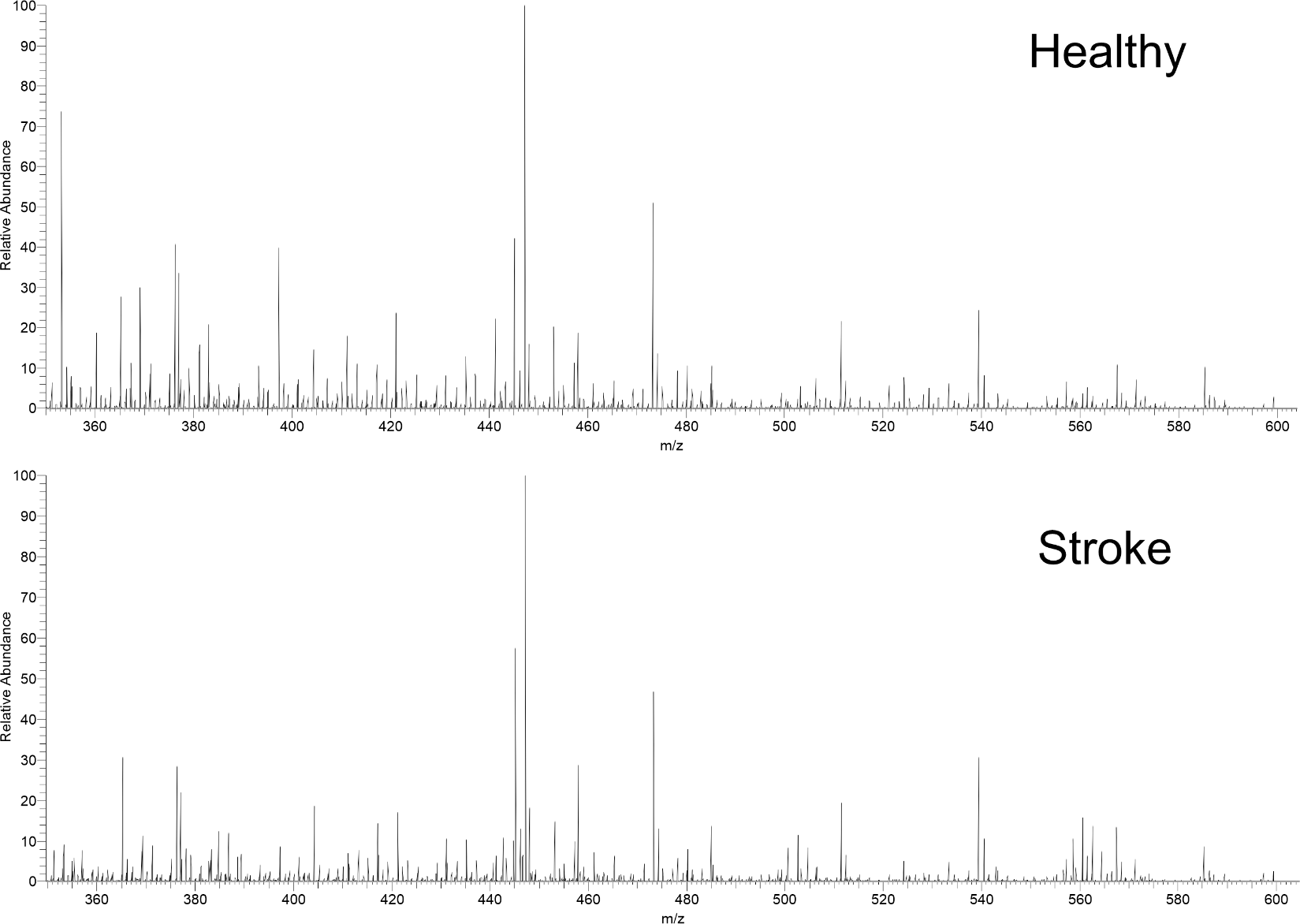


**Figure S2**. Mass spectra of healthy (top) and stroke (bottom) region of brain.


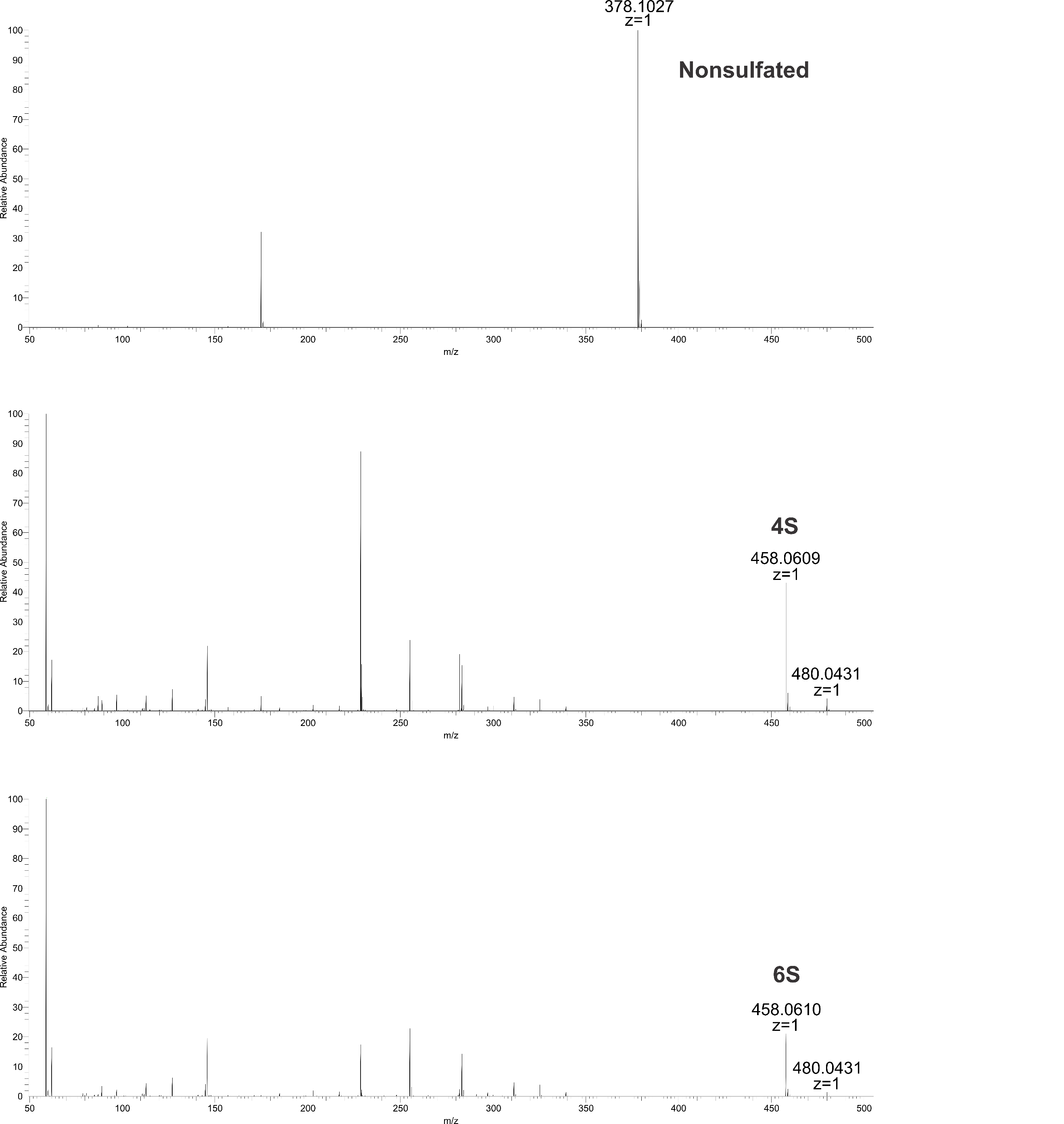


**Figure S3.** Mass spectra of non-sulfated (top), and mono-sulfated 4S-CS (middle) and 6S-CS (bottom) standards. 480 *m/z* is the sodium adduct peak.


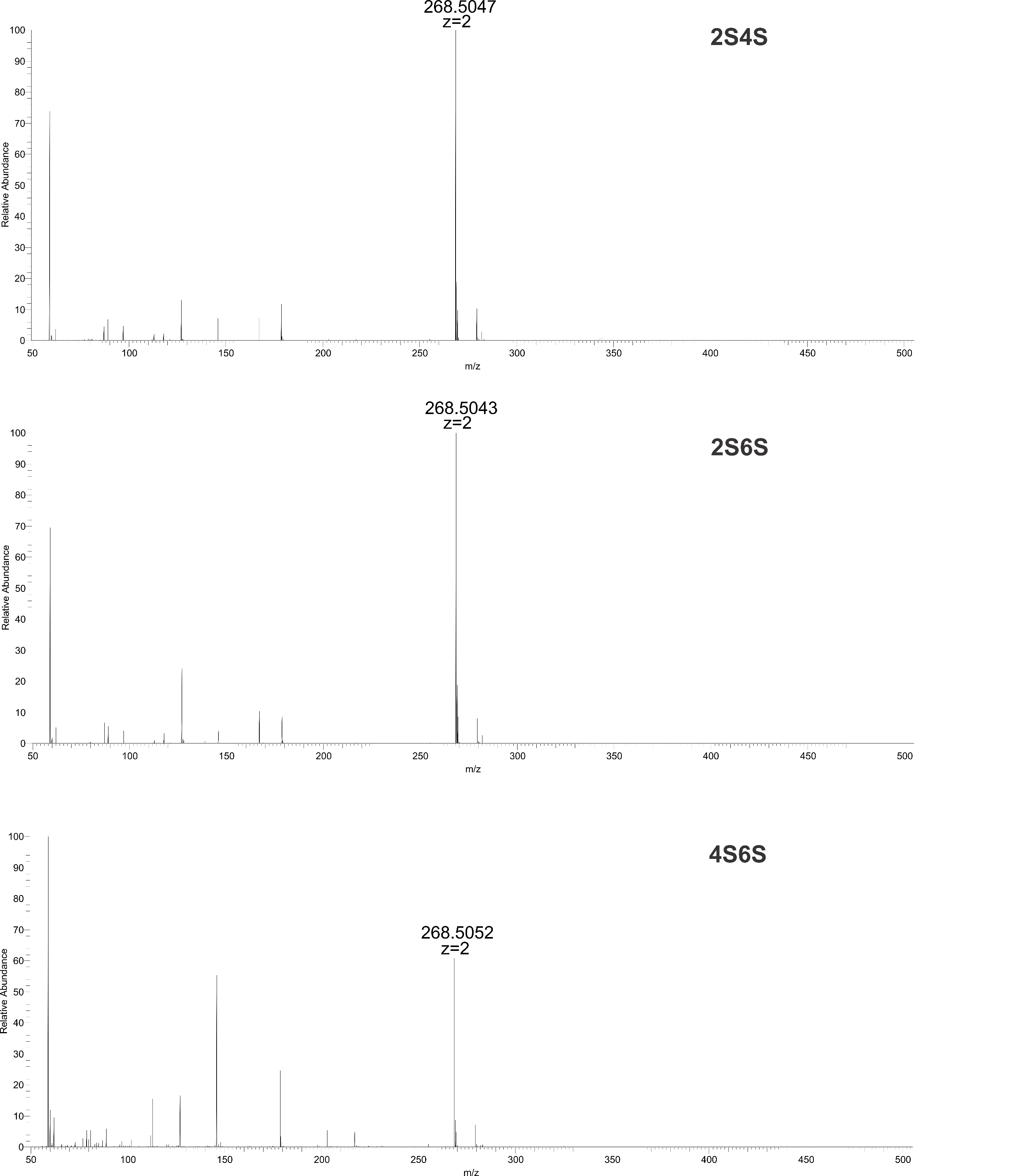


**Figure S4**. Mass spectra of di-sulfated 2S4S (top), 2S6S (middle), and 4S6S (bottom) standards.
